# Class III correction using an inter-arch spring-loaded module

**DOI:** 10.1186/s40510-014-0032-2

**Published:** 2014-05-02

**Authors:** Robert Vanlaecken, Michael O Williams, Thomas Razmus, Erdogan Gunel, Chris Martin, Peter Ngan

**Affiliations:** 1 Department of Orthodontics, West Virginia University School of Dentistry, 1073 Health Science Center North, Morgantown 26506, WV, USA; 2 Private practice, Watertown, SD, USA; 3 Private practice, Gulfport, MS, USA; 4 Department of Diagnostic Services, West Virginia University School of Dentistry, Morgantown, WV, USA; 5 Department of Statistics, West Virginia University, Morgantown, WV, USA

## Abstract

**Background:**

A retrospective study was conducted to determine the cephalometric changes in a group of Class III patients treated with the inter-arch spring-loaded module (CS2000®, Dynaflex, St. Ann, MO, USA).

**Methods:**

Thirty Caucasian patients (15 males, 15 females) with an average pre-treatment age of 9.6 years were treated consecutively with this appliance and compared with a control group of subjects from the Bolton-Brush Study who were matched in age, gender, and craniofacial morphology to the treatment group. Lateral cephalograms were taken before treatment and after removal of the CS2000® appliance. The treatment effects of the CS2000® appliance were calculated by subtracting the changes due to growth (control group) from the treatment changes.

**Results:**

All patients were improved to a Class I dental arch relationship with a positive overjet. Significant sagittal, vertical, and angular changes were found between the pre- and post-treatment radiographs. With an average treatment time of 1.3 years, the maxillary base moved forward by 0.8 mm, while the mandibular base moved backward by 2.8 mm together with improvements in the ANB and Wits measurements. The maxillary incisor moved forward by 1.3 mm and the mandibular incisor moved forward by 1.0 mm. The maxillary molar moved forward by 1.0 mm while the mandibular molar moved backward by 0.6 mm. The average overjet correction was 3.9 mm and 92% of the correction was due to skeletal contribution and 8% was due to dental contribution. The average molar correction was 5.2 mm and 69% of the correction was due to skeletal contribution and 31% was due to dental contribution.

**Conclusions:**

Mild to moderate Class III malocclusion can be corrected using the inter-arch spring-loaded appliance with minimal patient compliance. The overjet correction was contributed by forward movement of the maxilla, backward and downward movement of the mandible, and proclination of the maxillary incisors. The molar relationship was corrected by mesialization of the maxillary molars, distalization of the mandibular molars together with a rotation of the occlusal plane.

## Background

**Figure 1:**
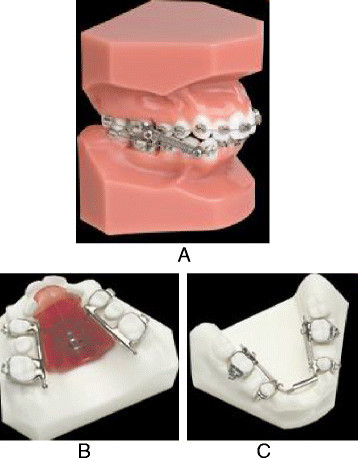
**The CS2000® appliance (A), TB SAG appliance (B), and MSX 2000 appliance (C)**.

Treatment of Class III malocclusions may include growth modification, camouflage with orthodontic tooth movement and orthognathic surgery [1]. In young patients with deficient maxilla, facemask is the appliance of choice whereas in patients with a normal maxilla and prognathic mandible, the chin cup appliance is usually preferred. In Class III patients with no growth remaining, fixed appliance with Class III elastics are usually used to camouflage the malocclusion [2]. However, most of these appliances require patient cooperation. If patients do not wear the appliance or elastics, treatment will fail. Fixed force module has been used in the correction of Class II malocclusion with the aim of reducing patient compliance [3,4]. The use of an inter-arch spring-loaded module to correct Class III malocclusion has not been reported in the literature. The CS 2000® appliance (Dynaflex, St. Ann, MO, USA) is a fixed spring-loaded module which has both upper and lower members. Depending on the patient's needs, the upper and lower appliances have differing components consisting of differing expansion components. The main components of these appliances are the inter-arch closed coil NiTi springs which are used in the same vector as Class III elastics (Figure 1).

The treatment response to Class III correctors has been reported extensively in the literature [5-12]. In a study by Tollaro, the mandibular retractor was able to rotate the mandible downward and backward to compensate for the excessive mandibular growth [5]. Baik found that the Frankel regulator III can correct Class III malocclusion in growing patients by a backward and downward rotation of the mandible and lingual tipping of the lower incisors [6]. Garattini used a Bionator III appliance to correct Class III malocclusion and concluded that the majority of changes can be attributed to dentoalveolar changes [9]. Similar results were noted by Kidner with the use of a Class III twin block appliance [8]. However, Proffit noted that these changes are not skeletal in origin, but mainly dentoalveolar [2]. These appliances allowed the maxillary molars to migrate mesially and hold the lower molars in place. They also proclined the upper incisors and retroclined the lower incisors, rotate the occlusal plane and/or the chin posterior, but have no major effect on the skeletal growth of the mandible or maxilla.

The facemask appliance has been used in the correction of Class III patients with maxillary deficiency [10,11],[13-18]. The goal of this appliance is to provide skeletal correction by protracting the maxilla and limiting the growth of the mandible. While this is thought to be the main effect, da Silva Filho also noted that this appliance also rotated the mandible down and back together with distalization of the mandibular teeth and mesialization of the maxillary teeth [13-16]. Ngan et al. reported on the treatment response of Class III patients to expansion and facemask therapy [10]. The overjet correction was attributed to a forward movement of the maxilla, backward rotation of the mandible, proclination of maxillary incisors, and retroclination of the mandibular incisors. Baccetti looked at how age affects treatment outcomes with a bonded RPE and facemask [11]. He found that in the early treatment group (6.8 ± 0.6 years), a significant forward movement of ‘A’ point occurred, while in the late treatment group (10.3 years ± 1.0 year), no significant A point movement was achieved. During post-treatment, Baccetti found that Class III growth patterns returned in the absence of any skeletal retention appliances [17]. Westwood also found a return to Class III growth patterns once treatment was complete and recommends an overcorrection during facemask treatment [18]. All of these appliances require significant patient compliance in order to achieve a reasonable treatment result. The objective of this study was to determine the cephalometric changes in a group of Class III patients treated with a fixed spring-loaded module that required minimal patient compliance (CS2000®, St. Ann, MO, USA) and compare the results with those reported by other Class III correctors.

## Methods

This study was approved by the Institutional Review Board of West Virginia University. Approval was also granted from one of the authors (MW) for the use of orthodontic records from his office. Seventy-five patients were treated consecutively by one of the authors (MW) with the CS2000® appliance. The inclusion criteria were that all subjects had no previous orthodontic treatment. All subjects were in the mixed to early permanent dentition ages. All subjects had a Class III molar occlusion or a mesial step and the pre-treatment Wits < 0. All subjects required comprehensive orthodontic treatment together with the CS2000® appliance. Patients with poor-quality radiographs or missing radiographs were excluded from the study. The final sample consisted of 30 patients (15 males and 15 females) with a mean age of 9.6 ± 2.1 years and a range of ages 6 to 15 years. The mean age for the male sample was 8.7 ± 1.7 years and the female sample, 10.4 ± 2.2 years. The mean treatment time was 1.3 ± 0.3 years. Lateral cephalograms were taken at pre-treatment (T1) and at completion of treatment with the CS2000® appliance (T2).

The control group consisted of serial cephalometric radiographs of 30 Class III subjects (15 boys, 15 girls) with no history of orthodontic treatment from the Bolton-Brush Study. The control subjects were closely matched in age, sex, and craniofacial morphology with the experimental subjects (Table 1). Significant differences were found in 6 of the 26 cephalometric variables indicating that the starting form of the treatment group has a more forward position of the mandible and the control group has greater increase in the lower facial height and mandibular plane angle.

**Table 1:** Comparison of pre-treatment craniofacial morphology of control and treatment samples

**Variable**	**Control**		**Treated**		**Diff**	***p*** **value**	**Sig**
	**Mean**	**SD**	**Mean**	**SD**			
Sagittal							
OLp-A point	67.78	3.61	68.31	2.71	0.52	0.53	NS
OLp-B point	71.87	3.81	74.28	3.38	2.41	0.01	S
OLp-Pg	74.15	4.34	76.70	4.13	2.54	0.02	S
OLp-Co	−9.74	2.09	−10.06	3.69	−1.32	0.09	NS
Wits	−3.62	3.77	−4.22	2.14	−0.60	0.45	NS
Is-OLp	73.89	4.73	74.13	3.89	0.23	0.83	NS
Ii-OLp	73.20	3.92	74.12	3.85	0.92	0.36	NS
Overjet	0.69	2.43	0.01	2.29	0.68	0.26	NS
Ms-OLp	45.94	3.17	47.05	3.36	1.11	0.19	NS
Mi-OLp	49.24	3.08	49.63	3.19	0.38	0.63	NS
Molar relationship	−3.30	1.82	−2.57	1.92	0.72	0.13	NS
Vertical							
OLs-A point	27.04	3.92	29.40	5.22	2.36	0.06	NS
ANS-Me	59.34	4.22	56.40	4.96	−2.93	0.01	S
Is-NL	24.36	2.32	23.41	2.23	−0.85	0.15	NS
Ii-ML	34.10	3.07	34.05	2.48	−0.05	0.94	NS
Overbite	1.57	1.29	2.31	1.54	0.74	0.06	NS
Msc-NL	17.81	1.68	18.77	2.41	0.96	0.07	NS
Mic-ML	25.70	2.37	24.77	2.34	−0.93	0.11	NS
Angular							
SNA	79.65	3.91	80.07	3.80	0.42	0.67	NS
SNB	77.39	2.77	80.39	3.59	3.09	0.01	S
ANB	2.36	3.32	−0.29	1.61	−2.66	0.01	S
SNL-NL	9.64	2.67	9.01	3.30	−0.63	0.41	NS
SNL-ML	38.83	4.97	32.81	5.01	−6.02	0.01	S
SNL-OLs	21.92	3.59	20.27	3.75	−1.65	0.06	NS
Is/SNL	100.17	6.73	102.24	9.02	2.07	0.31	NS
Ii/ML	86.65	6.73	85.81	7.16	−0.84	0.64	NS
Interincisal angle	134.20	7.68	139.12	13.30	4.92	0.08	NS

Overjet (Is/OLp − Ii/OLp), molar relationship (Ms/OLp − Mi/OLp), maxillary base (A/OLp), mandibular base (pg/OLp), maxillary incisor (Is/OLp), mandibular incisor (Ii/OLp), maxillary molar (Ms/OLp), and mandibular molar (Mi/OLp).

The CS 2000® appliance is a fixed Class III corrector consisting of an upper member, the Tooth Born Sagittal (TB SAG) appliance, and a lower member, the MSX 2000 (Dynaflex, St. Ann, MO, USA), and an inter-arch NiTi springs from upper-first molars to lower-first bicuspids (Figure 1). The TB SAG is the upper member of the CS2000® consisting of NiTi expansion springs for transverse correction as well as NiTi springs in an anterior-posterior direction that places a protraction force on the pre-maxilla. It also provides an attachment at the first molars for NiTi springs. The MSX 2000 is the lower member of the CS2000® appliance that provides mandibular arch transverse correction with NiTi springs. It also provides an attachment at the first bicuspids for NiTi springs. Depending on the patient's needs, the upper and lower appliances have differing components consisting of differing expansion components. The main components of these appliances are the inter-arch closed-coil NiTi springs in the same vector as Class III elastics. When the inter-arch spring module is attached to the pivot teeth, it will cause these teeth to resist the force expressed by the 150-g NiTi coil spring on each side. When the pivot teeth are coupled with the 300-g coil springs, they will reinforce and serve as the anchorage teeth. This principle allows the inter-arch spring module to be a totally intraoral anchorage appliance and does not rely on the forehead and chin as anchorage as in the case of protraction facemask therapy. Therefore, this appliance requires minimal patient compliance.

### Cephalometric analysis

**Figure 2:**
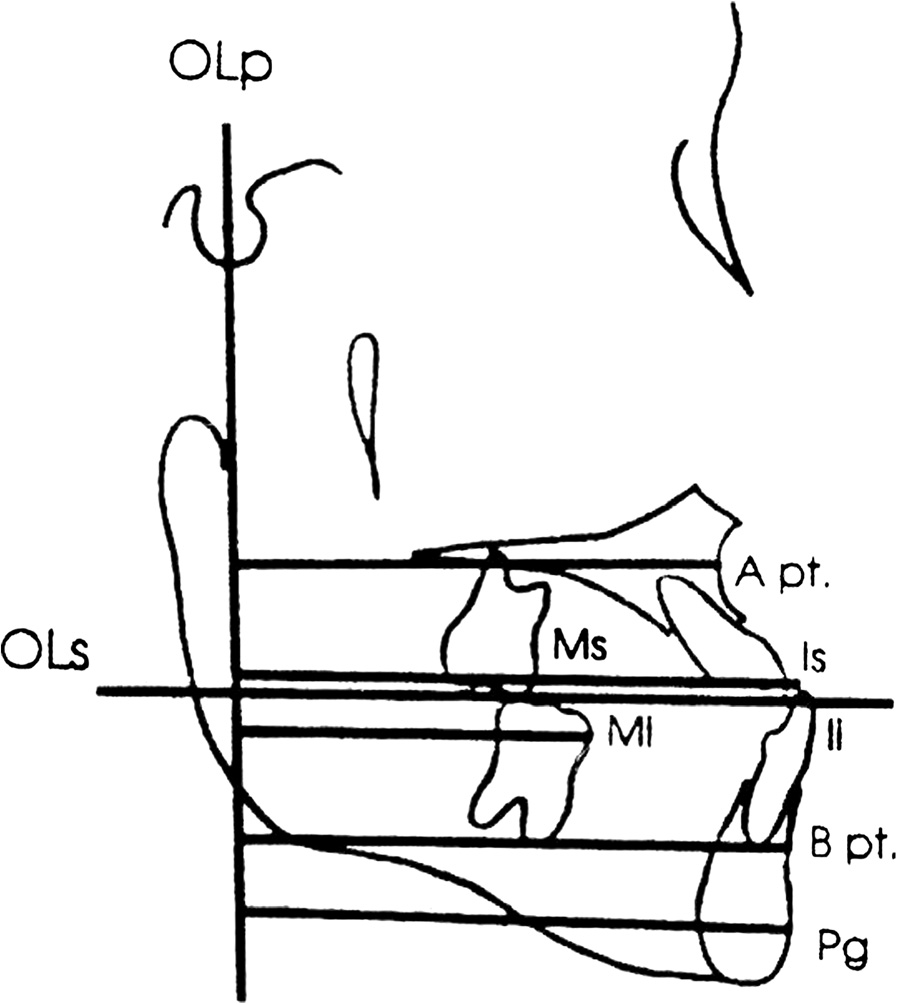
Cephalometric landmarks and lines used for sagittal measurements.

**Figure 3:**
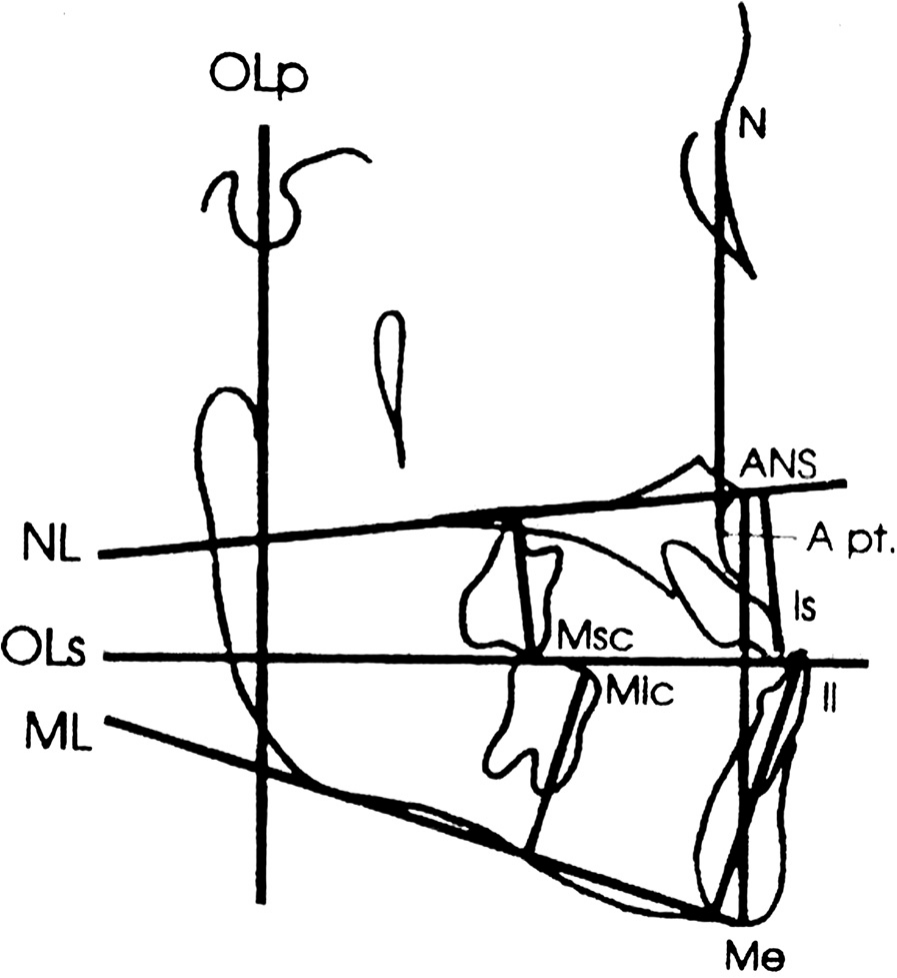
Cephalometric landmarks and lines for vertical measurements.

**Figure 4:**
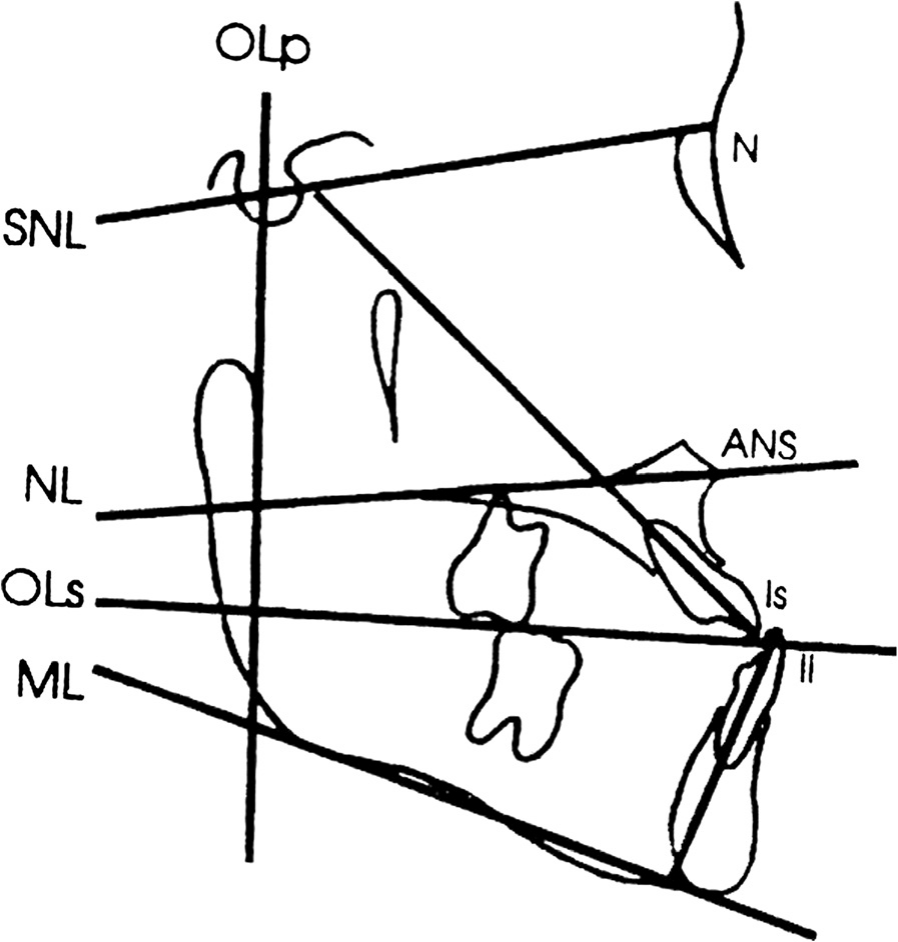
Cephalometric landmarks and lines for angular measurements.

Pre- and post-treatment lateral cephalograms from both groups were digitized using the Dolphin Imaging (Dolphin Imaging, Chatsworth, CA, USA) software to allow for landmark identification and adjusting for magnification. The cephalometric systems described by Bjork [19] and Pancherz [20] were used to analyze the treatment changes. The landmarks used are shown in Figures 2, 3, and 4. The magnification factor of the lateral cephalograms was found to be 6% both for the control and treated subjects. The angular measurements were located using the Dolphin Imaging system (Dolphin Imaging, Chatsworth, CA, USA) and reported to the nearest 0.1°. Films were printed 1:1 using a Kodak ESP 7250 printer (Kodak, Atlanta, GA, USA), and then traced by one investigator using a #2 lead pencil on 0.003 in. matt cephalometric acetate tracing film (3 M Unitek, Monrovia, CA, USA). All linear measurements were performed using a digital caliper (accurate to 0.01 mm) and reported to the nearest 0.1 mm. Analysis of the sagittal skeletal and dental changes were recorded along the occlusal plane (OLs) and to the occlusal plane perpendicular (OLp) from the first cephalogram; this formed the reference grid. The grid was then transferred to subsequent cephalograms by superimposing the tracing on the mid-sagittal cranial structures. The changes in overjet and molar relationship were calculated using the formula depicted in Table 3.

### Data analysis

The dentofacial morphology of the subjects in the experimental group was compared using paired *t* tests. The starting forms of the control and experimental samples were compared with a two-tailed *t* test. The skeletal and dental changes between the treated and control sample at the two time periods were compared with a two-tailed *t* test. The confidence level was set at 95%.

### Method error

The error in locating, superimposing, and measuring the changes of the landmarks by one examiner were measured on the cephalograms of 10 randomly selected subjects. All cephalograms were recorded twice independently on two separate occasions with a 2-week interval. For all the cephalometric variables, differences between the independent repeated measurements of each individual before/after treatment were recorded. The intraclass correlation coefficient of reliability (*R*) was used to determine the reliability of cephalometric measurements. The *R* value ranged from 0 to 1.00, with *R* value greater than 0.90 indicating high reliability. The mean differences for all linear measurements were less than 0.8 mm. The greatest mean error for angular measurement was 0.9° for the measurement of maxillary central incisal angle (Is/NSL) and mandibular central incisal angle (Ii/ML).

## Results

### Cephalometric changes

Changes in cephalometric measurements in patients treated with the CS2000® appliance (T2-T1) are shown in Tables 2, 3, and 4. The appliance effects were calculated by subtracting the changes due to growth from the treatment changes.

**Table 2:** Individual sagittal changes (mm) between pre-treatment and post-treatment in 30 subjects

	**Overjet**	**Maxillary incisor**	**Mandibular incisor**	**Molar relationship**	**Maxillary molar**	**Mandibular molar**	**Maxillary base**	**Mandibular base**
Patient								
1	4.7	3.7	−1.0	5.4	4.5	−0.9	1.3	0.1
2	0.8	4.7	3.9	−1.0	−2.6	−1.6	0.3	−1.4
3	−7.7	−5.4	2.3	11.8	4.1	−7.7	0.5	−5.3
4	0.4	1.0	0.6	7.5	2.7	−4.8	0.9	−1.0
5	6.0	−0.3	−6.3	6.2	−0.5	−6.7	−0.4	−3.0
6	11.5	3.8	−7.7	7.8	0.9	−6.9	0.9	−6.2
7	8.9	4.9	−4.0	6.4	2.4	−4.0	0.6	−1.8
8	0.6	−0.7	−1.3	7.7	3.6	−4.1	1.3	−2.5
9	9.8	5.0	−4.8	6.5	−1.6	−8.1	−0.1	−4.4
10	7.0	6.0	−1.0	9.4	2.8	−6.6	1.1	−1.9
11	2.1	2.2	0.1	1.8	1.3	−0.5	0.2	−2.6
12	5.1	2.0	−3.1	7.7	3.5	−4.2	0.5	−4.8
13	7.8	3.9	−3.9	−4.8	−3.5	1.3	−0.8	−4.9
14	4.2	2.1	−2.1	3.0	1.2	−1.8	0.0	−2.5
15	−0.5	4.0	4.5	5.9	2.7	−3.2	3.0	−0.2
16	0.2	−1.1	−1.3	0.4	−1.1	−1.5	0.4	−2.6
17	1.0	2.1	1.1	3.1	0.9	−2.2	1.6	−2.0
18	6.1	3.9	−2.2	10,6	6.6	−4.0	1.2	−2.2
19	5.3	1.6	−3.7	2.2	3.1	0.9	−0.4	−4.1
20	4.2	−0.1	−4.3	4.8	1.2	−3.6	1.3	−4.6
21	10.0	2.3	−7.7	9.1	1.8	−7.3	1.2	−7.5
22	11.3	6.1	−5.2	1.2	−0.2	−1.4	0.9	−3.0
23	7.4	8.5	1.1	7.1	6.3	−0.8	4.5	0.7
24	5.0	4.1	−0.9	6.1	4.7	−1.4	1.5	0.4
25	2.8	−4.5	−7.3	5.9	2.3	−3.6	−1.4	−5.5
26	3.4	4.0	0.6	7.0	4.1	−2.9	0.8	−0.6
27	0.4	3.2	2.8	−3.5	−0.7	2.8	0.7	2.1
28	5.8	−2.5	−8.3	7.4	0.0	−7.4	−0.4	−7.4
29	4.4	2.9	−1.5	8.4	2.9	−5.5	0.7	−2.6
30	−8.3	−3.0	5.3	4.2	0.5	−3.7	1.4	−3.4
Pooled								
Mean	4.0*	2.1*	−1.8*	5.2*	1.8*	−3.4*	0.8*	−2.8*
SD	4.7	3.2	3.7	3.9	2.4	2.8	1.1	3.7
Min	−8.3	−5.4	−8.3	−4.8	−3.5	−8.1	−1.4	−8.3
Max	11.5	8.5	5.3	11.8	6.6	2.8	4.5	5.3

Overjet (Is/OLp − Ii/OLp), molar relationship (Ms/OLp − Mi/OLp), maxillary base (A/OLp), mandibular base (Pg/OLp), maxillary incisor (Is/OLp), mandibular incisor (Ii/OLp), maxillary molar (Ms/OLp), and mandibular molar (Mi/OLp).

* = p<.05.

**Table 3:** Individual vertical changes (mm) between pre-treatment and post-treatment in 30 subjects

	**Maxillary base**	**Overbite**	**LFH**	**Maxillary incisor**	**Mandibular incisor**	**Maxillary molar**	**Mandibular molar**	**NL/SNL**	**ML/SNL**	**OL/SNL**
Patient										
1	2.2	−2.0	0.7	3.4	−3.6	4.1	−2.5	−1.4	−1.9	0.0
2	1.5	−4.0	5.3	2.1	0.5	−1.8	2.3	−2.7	3.2	−1.4
3	0.2	−2.4	6.0	2.4	1.6	1.7	−0.9	−3.5	3.8	0.2
4	1.9	−2.7	4.0	−0.3	1.9	1.4	−0.4	−3.2	0.7	1.0
5	0.6	−0.3	2.5	−2.7	2.0	0.1	−1.8	0.4	1.7	−0.2
6	−2.3	−5.7	4.8	−3.4	−1.2	2.8	−2.4	−0.3	3.7	−0.3
7	−0.2	−1.6	3.5	1.5	−1.1	1.4	−0.7	−3.3	2.8	0.6
8	1.6	−0.1	5.3	3.0	1.9	−0.7	−0.6	−8.4	−1.1	−0.5
9	0.2	−3.4	3.6	−0.7	−0.6	1.1	−1.4	−5.8	1.4	−3.0
10	−0.2	−1.1	7.2	0.4	3.3	4.6	−1.6	−2.2	3.0	1.0
11	−1.1	−1.3	4.8	2.6	−0.2	0.0	1.1	−6.6	2.1	−1.2
12	0.7	−1.4	6.6	−0.7	4.5	3.6	0.3	−3.9	0.1	−3.3
13	−0.7	−2.3	4.4	−2.7	3.2	−0.5	2.2	−1.8	5.3	0.1
14	−1.0	−5.2	5.5	−0.3	−0.6	−1.3	3.9	−4.2	3.0	0.3
15	2.5	−1.2	3.0	0.7	3.2	2.1	0.8	0.7	0.8	0.3
16	−0.1	1.2	5.1	4.0	1.9	2.2	0.9	−0.1	2.9	−0.6
17	1,8	−2.3	4.1	−4.2	5.2	0.1	1.8	−3.1	−1.3	0.3
18	0.1	−1.4	5.3	2.6	0.5	4.5	−0.6	0.4	4.9	−0.6
19	−0.2	−5.1	5.5	−5.3	1.4	0.5	1.9	−3.4	−0.2	−1.3
20	0.6	−0.6	4.6	1.8	1.9	3.3	−0.7	−3.2	3.3	−3.6
21	2.2	−4.6	1.8	−4.1	1.0	−1.3	0.3	−5.7	−1.2	−3.1
22	0.8	1.9	2.6	1.4	2.2	0.5	−0.5	−1.9	2.1	−1.2
23	2.9	1.9	6.4	−1.3	3.2	6.1	−1.3	1.1	2.8	−0.7
24	2.0	−0.2	2.5	1.0	1.2	3.7	−1.7	−1.6	0.7	−0.7
25	−0.9	−0.8	2.7	−0.5	−0.4	0.9	−0.9	−2.0	4.8	0.1
26	1.2	−1.0	4.8	−0.7	4.3	3.3	−1.5	−1.7	0.8	0.3
27	−1.2	−2.9	3.7	0.5	0.7	−1.2	1.9	−5.6	−2.4	−1.0
28	−1.2	−3.3	2.7	−2.8	0,9	0.0	−0.7	−1.6	3.2	−0.5
29	−0.7	−4.0	3.0	−1.2	0.1	2.9	−1.7	−2.7	−0.7	−0.9
30	2.1	−2.3	4.1	1.1	2.5	1.8	2.6	−2.6	−1.2	−1.1
Pooled										
Mean	0.5*	−1.9*	4.2*	−0.1*	1.4*	1.5*	−0.1	−2.7*	1.6*	−0.7*
SD	1.3	2.0	1.5	2.4	1.9	2.0	1.7	2.2	2.2	1.2
Min	−2.3	−5.7	0.7	−5.3	−3.6	−1.8	−2.5	−8.4	−2.4	−3.6
Max	2.9	1.9	7.2	4.0	5.2	6.1	3.9	1.1	5.3	1.0

* = p<.05.

**Table 4:** **Individual angular changes (**°**) between pre-treatment and post-treatment in 30 subjects**

	**SNA**	**SNB**	**ANB**	**Maxillary incisor**	**Mandibular incisor**	**Interincisal angle**	**OLp-Co**	**Wits**
Patient								
1	1.1	0.1	1.0	4.9	6.0	−9.0	1.4	1.8
2	0.0	−1.1	1.0	9.4	15.6	−28.3	4.7	−3.7
3	2.2	−3.0	5.2	−9.2	−4.6	8.7	0.0	5.6
4	−0.2	−0.7	0.5	0.1	5.5	−6.4	1.3	2.9
5	1.1	−0.4	1.6	14.8	−11.3	−5.4	2.4	5.4
6	2.9	−3.2	6.1	17.7	−6.8	−14.4	−1.0	9.9
7	1.2	−1.9	2.8	10.1	−3.7	−9.2	2.3	3.2
8	3.5	1.1	2.4	−8.3	−2.4	11.7	1.8	3.0
9	5.7	1.2	4.6	20.3	−1.6	−20.2	3.3	6.2
10	1.7	−1.2	2.9	16.0	−2.8	−16.1	0.3	6.9
11	2.8	0.4	2.4	−2.5	6.5	−6.1	4.0	0.1
12	5.0	1.2	3.7	15.1	8.9	−24.2	1.3	8.7
13	0.6	−2.9	3.4	23.6	−3.9	−24.8	0.8	8.0
14	1.6	−1.0	2.6	7.4	−0.9	−9.5	4.5	−1.0
15	1.8	−0.7	2.3	2.2	10.3	−13.4	−2.4	2.4
16	1.8	0.5	1.3	−4.1	−1.3	2.6	4.3	0.0
17	3.2	0.2	2.9	10.7	0.0	−9.5	−0.1	8.5
18	−2.4	−3.9	1.5	9.6	−6.7	−7.8	3.9	3.8
19	2.2	0.2	1.9	17.6	−0.5	−18.5	2.2	6.6
20	5.6	0.6	5.1	−7.5	−7.1	11.7	0.2	6.7
21	4.9	−1.7	6.6	10.6	−6.8	−2.5	−2.9	11.6
22	0.9	−1.4	2.2	20.4	−4.2	−18.4	0.8	4.2
23	2.6	−0.7	3.3	6.6	−3.8	−5.6	−1.2	8.7
24	−0.3	0.3	−0.6	16.2	−6.7	−10.0	−3.5	3.9
25	−3.1	−4.7	1.5	−5.0	−6.0	6.1	3.7	4.9
26	1.6	−0.3	2.1	18.3	3.0	−22.1	1.4	7.6
27	2.5	3.2	−0.7	10.2	5.0	−12.9	1.5	−1.2
28	0.9	−3.3	4.2	3.0	−5.4	−0.3	1.3	7.3
29	3.2	1.1	1.9	15.2	−2.3	−12.1	0.6	4.6
30	3.0	0.2	2.8	−0.4	−0.1	0.3	−0.3	3.2
Pooled								
Mean	1.9*	−0.7*	2.6*	8.1	−0.9	−8.9*	1.2*	4.7*
SD	2.0	1.8	1.7	9.4	6.1	10.6	2.1	3.6
Min	−3.1	−4.7	−0.7	23.6	−11.3	−28.3	−3.5	−3.7
Max	5.7	3.2	6.6	−9.2	15.6	11.7	4.7	11.6

* = p<.05.

#### Sagittal differences

**Figure 5:**
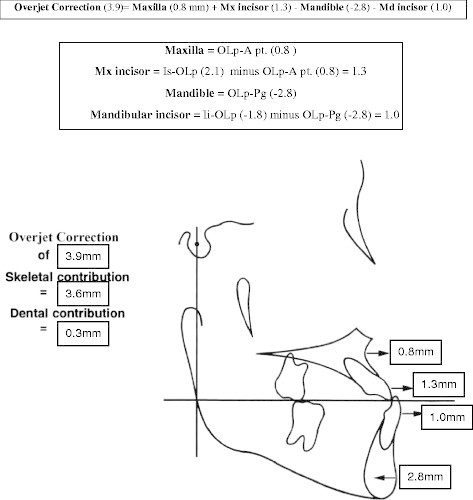
Skeletal and dental contributions to overjet correction (T2-T1).

**Figure 6:**
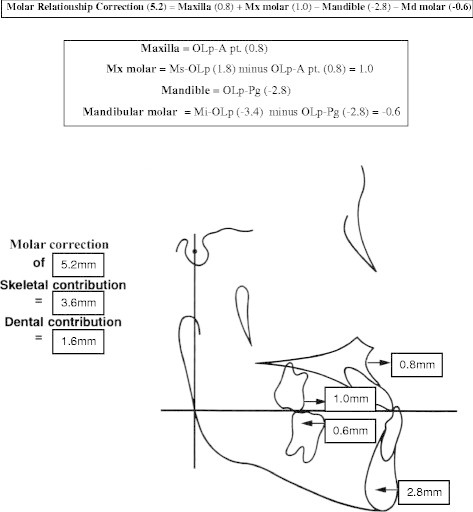
Skeletal and dental contributions to molar relationship correction (T2-T1).

Significant differences were found in all the sagittal variables measured (Table 2). Figures 5 and 6 summarize the skeletal and dental contributions to the overjet and molar corrections from treatment. With an average treatment time of 1.3 years, all subjects were corrected to a Class I or overcorrected to a Class I or II dental arch relationship. Overjet and sagittal molar relationships improved by an average of 3.9 and 5.2 mm, respectively. This was a result of a 0.8-mm forward maxillary movement, a 2.8-mm backward movement of the mandible, a 1.3-mm labial movement of the maxillary incisors, a 1.0-mm labial movement of the mandibular incisors, a 1.0-mm mesial movement of the maxillary molar, and a 0.6-mm distal movement of the mandibular molars. The relationship of the maxillary base to the mandibular base relative to the functional occlusal plane (Wits) for the treatment group (T2-T1) was found to be increased by 4.7 mm after subtracting out the control group. As for individual sagittal changes, with 1.3 years of inter-arch treatment with the force module, the mean overjet changes were large (4.0 mm) and the variations were wide ranging from −8.3 to 11.5 mm. In general, the effect of the force module on the maxillary base was small with a mean increase of 0.8 mm, but the variation was wide, ranging from −1.4 to 4.5 mm. The sagittal change in the mandibular base was larger with a mean decrease of 2.8 mm, ranging from −8.3 to 5.3 mm. Large variations were also found with changes in the maxillary incisors, mandibular incisors, maxillary molars, and mandibular molars.

#### Vertical differences

Significant differences were found in all vertical measurements except Mic-ML (Table 3). The maxillary base (OLs-A point) was found to move inferiorly by 0.5 mm. The lower facial height ANS-Me increased by 4.2 mm. The maxillary incisor (Is-NL) intruded by 0.1 mm as compared to a reference line from ANS-PNS. The mandibular incisor (Ii-ML) extruded by 1.4 mm from the mandibular plane (Go-Me) and was significant. During treatment, overbite decreased by 1.9 mm. The maxillary molar (Msc-NL) erupted by 1.5 mm, while the mandibular molar (Mic-ML) remained relatively unchanged, intruding by 0.1 mm which was found to be non-significant. With 1.3 years of treatment with the force module, vertical maxillary base change was small with an average of 0.5 mm with a range of −2.3 to 2.0 mm. The overbite reduction in individual subjects ranged from −5.7 to 1.9 mm. The lower face height increased in all subjects. No consistent pattern was found in vertical changes of incisors and molars.

#### Angular differences

Significant differences were found between all angular measurements except Is/NL and Ii/ML (Table 4). SNA increased by 1.9° during treatment and was significant, while SNB remained decreased at 0.7°. ANB thus increased by 2.6° during treatment and was found significant. The Is-NL was found to procline at 8.1° during treatment and the mandibular incisor retroclined at 0.9° during treatment in relation to the mandibular plane (Go-Me), but were neither significant. The inter-incisal angle was found to increase by 8.9°. NL to SN was found to decrease by 2.7° during treatment indicating a counterclockwise rotation of the palatal plane. ML to SN was found to increase by 1.6°, indicating a clockwise rotation of the mandibular plane. The OL to SN was found to decrease by 0.7° during treatment, indicating a counterclockwise rotation of the occlusal plane.

## Discussion

The use of an inter-arch spring-loaded module (CS2000®) to correct Class III malocclusion has not been reported in the literature. However, different treatment modalities have been used to treat Class III malocclusions ranging from protraction facemasks [10,15,21], to removable appliances such as the Frankel III [8,9], Bionator III [10], modified tandem traction bow [12], and Class III Twin Block [11], to inter-arch protraction springs described by Liou [21,22]. With the increase in popularity of skeletal anchorage devices such as miniscrews and miniplates [23,24], more treatment possibilities will be available.

### Sagittal differences

In the present study, significant changes were found in all the sagittal variables as compared to the control group. The maxilla (A point) was found to move forward by 0.8 mm over a period of 1.3 years. In a study with protraction facemask [10], A point was found to move forward by an average of 1.8 mm over a 6-month period. Baccetti found 2.3-mm- and 3.1-mm-forward movements of A point in young patients treated using protraction facemasks [11,17]. Most of the studies reported forward movements ranging from 1.5 to 3.4 mm [13-15]. Loiu reported a 5.8-mm-forward movement of the maxilla in 3 months using a maxillary expansion and constriction protocol in conjunction with protraction facemask [25]. This is because the expansion protocol allows loosening of the maxillary sutures and the protraction spring acts on the sutures 24 h per day. The correction using the inter-arch spring-loaded module also acts on the maxillary sutures full time. However, the force magnitude (150 g per side) is smaller than those exerted by the facemask (450 g per side). Therefore, the results were comparable to those reported using removable appliances. Atalay found an increase in the length of the maxilla (Co-A point) of 1.8 mm using the tandem traction bow appliance [12]. Baik et al. reported a forward movement of 1.3 mm with the FRIII removable functional appliance over 1.3 years of treatment [8]. The inter-arch spring module, when attached to the pivot teeth, will cause these teeth to resist the force expressed by the 150-g NiTi coil spring on each side. When the pivot teeth are coupled with the 300-g coil springs, they will reinforce and serve as the anchorage teeth. This principle allows the inter-arch spring module to be a totally intraoral anchorage appliance and does not rely on the forehead and chin as anchorage as in the case of protraction facemask therapy. Therefore, this appliance requires minimal patient compliance.

The mandibular base was found to move posteriorly by 2.8 mm partially due to a downward and backward rotation of the mandible as evidenced by a 4.2-mm change in lower facial height (ANS-Me) and an increase in the mandibular plane angle of 1.6°. Baccetti also reported a rotation of the mandible and a 2.5-mm restriction in mandibular protrusion with protraction facemask [11,17]. Ngan reported a 2.5-mm posterior movement of the mandibular base and a 2.9-mm increase in the lower facial height. [10] Baik, also showed a new backward movement of the mandibular base by 2.5 mm with the use of removable FRIII appliance [6].

The Wits measurements were found to improve by 4.7 mm. This result is similar to those reported using the protraction facemask [10], but more than the 2.7 mm reported using the FRIII appliance [6] and the 2.4 mm by the Bionator III appliance [9]. This change can be partially attributed to a change in the occlusal plane rotation, as its inclination decreased in the treatment group (−1.9°) as referenced from SNL. The palatal plane also rotated in a counterclockwise direction as its angle decrease 2.2 mm throughout treatment as well. Similar change in occlusal and palatal planes rotating counter clockwise with treatment are seen with protraction facemask treatment [15]. Liou also reported a counterclockwise rotation of the maxilla and clockwise rotation of the mandible with the maxillary expansion and constriction protocol [22].

In the present study, the overjet was corrected by an average of 3.9 mm. This is less than those reported using the protraction facemask [10,15], but similar to those using removable appliances due to the differences in force value [5-9]. Ninety-two percent of the change was contributed by forward movement of the maxilla and backward movement of the mandible. The other 8% was attributed to the forward movement of the maxillary incisor (1.3 mm). This is helpful for the stability of Class III correction to maximize the skeletal changes and minimize dental changes. As for the lower incisors, most of the studies with removable appliances reported a backward movement of the lower incisors [5-9]. In the present study, the lower incisors were found to move forward by an average of 1 mm indicating that the use of the MSX 2000 appliance in the lower arch with the coil spring was able to minimize the side effect of proclining the lower incisors.

In the current study, the average molar relationship was corrected by 5.2 mm. This was similar to those reported by the protraction facemask studies [10,18,25,26]. However, only 69% of the correction was attributed to skeletal change. The rest were due to forward movement of the maxillary molars indicating a loss of anchorage in the upper arch, but may be helpful in the correction of molar relationship. Similarly, the lower molars in the current study were found to move forward by 1.4 mm instead of backward as reported by other studies [10,18,25,26].

### Vertical differences

In the present study, the overbite was decreased by 1.5 mm. This bite opening effect was similar to those noted in the facemask [10,18,25,26] and FRIII studies [6,7] and is mostly likely attributed by the vertical movement of the maxilla and downward and backward rotation of the mandible together with the changes in the dentition. The maxillary base was found to move inferiorly by 0.5 mm. The maxillary incisor intruded by 0.1 mm while the maxillary molar extruded by 1.5 mm. This then results in a counterclockwise rotation of the maxillary base, which is common with Class III correction modalities. The mandibular molar remained relatively unchanged, while the mandibular incisor extruded by 1.4 mm. This extrusion of the maxillary molar, without any compensation seen from the lower posterior dentition (intrusion) or significant vertical growth of the mandible, could have played a major role in the mandibular body's back and downward rotation, and the increase seen in lower facial height, as discussed earlier. In conjunction with the lower incisor and maxillary molar extrusion and the maxillary incisor remaining relatively in the same location, the occlusal plane as a unit decreased in angulation in reference to SNL. This result then attributes, in part, to the improvements seen in Wits measurements.

### Limitation of the study

Readers should be cautious; this study is limited to only 1.3 years of evaluation of this inter-arch spring-loaded module. Long-term follow-up of these patients will elucidate the stability of this treatment modality.

## Conclusions

Patients with a mild to moderate Class III malocclusion can be corrected using the inter-arch spring-loaded appliance with minimal patient compliance. The overjet correction was contributed by forward movement of the maxilla, backward and downward movement of the mandible, and proclination of the maxillary incisors. The molar relationship was corrected by mesialization of the maxillary molars and distalization of the mandibular molars, together with rotation of the occlusal plane.

## Competing interests

The authors declared that they have no competing interests.

## Authors’ contributions

RV carried out the study; MW provided the sample for the study; TR provided input to the cephalometric measurements; EG provided statistical analysis, CM provided the sequence of the study, PN drafted the manuscript. All authors read and approved the final manuscript.
